# Estimation of the prevalence of thyroid dysfunction in Catalonia through two different registries: Pharmaceutical dispensing and diagnostic registration

**DOI:** 10.1002/edm2.167

**Published:** 2020-07-08

**Authors:** Sara Torrejón, Lluis Vila, Berta Soldevila, Montse Martín, Manel Puig‐Domingo

**Affiliations:** ^1^ Endocrinology and Nutrition Department Hospital Sant Joan Despí Moisès Broggi Sant Joan Despi Spain; ^2^ Endocrinology and Nutricion Department Hospital Germans Trias I Pujol Badalona Spain; ^3^ Epidemiology Department Hospital Sant Joan Despí Moisès Broggi Sant Joan Despi Spain

**Keywords:** epidemiology, ICD‐9 diagnostic codes, pharmacoepidemiology, thyroid dysfunction

## Abstract

**Background:**

Population studies on the prevalence of thyroid dysfunctions are costly. The pharmacy dispensing (PDR) and diagnosis (DR) registers allow us to study the epidemiology of these pathologies in a simpler way. Our aims: 1/Estimate the prevalence of thyroid dysfunction in Catalonia based on data from the PDR and the DR, 2/to evaluate the concordance of the results obtained by both strategies.

**Methods:**

The population studied was the one registered with the public health system in Catalonia(Catsalut). In the PDR analysis, the information obtained through the Pharmaceutical Provision file (during 2012, 2013, 2014) was used regarding the number of patients under treatment (NPT) (levothyroxine and antithyroid medication). The DR analysis (2014) was performed by ICD‐9 codes (hyperthyroidism 242 and hypothyroidism 243, 244).

**Results:**

According to the NPT in the PDR analysis, the prevalence of treated hypothyroidism increased over 3 years: 2.81%(2012), 2.92%(2013) and 3.07%(2014) (*P* < .00001). The prevalence of hyperthyroidism in treatment was 0.14%(2012), 0.13%(2013) and 0.14%(2014). According to the DR analysis in 2014, the prevalence of hypothyroidism was 2.54% and 0.35% for hyperthyroidism. The PDR analysis estimated a higher hypothyroidism prevalence compared to that estimated by the DR (*P* < .0001) and vice versa in the case of hyperthyroidism.

**Conclusion:**

Both PDR and DR prevalence estimations of thyroid dysfunction show some degree of discordance probably due to undercoding bias in the case of DR and the absence of subclinical pathology in the case of PDR. However, both approaches are valid and complementary for estimating the prevalence of thyroid dysfunction.

## Graphical abstract

To Estimate the prevalence of thyroid dysfunction in Catalonia based on data from the pharmacy dispensing (PDR) and diagnosis registers (DR). The PDR analysis estimated a higher hypothyroidism prevalence compared to that estimated by the DR and vice versa in the case of hyperthyroidism. Although both PDR and DR prevalence estimations of thyroid dysfunction show some degree of discordance, both approaches are valid and complementary for estimating the prevalence of thyroid dysfunction.

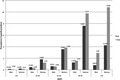

## 1 INTRODUCTION

Thyroid dysfunction is one of the most frequent endocrine disorders. However, there are few studies on its prevalence due to the high cost of conducting large‐scale cross‐sectional epidemiological studies. In addition, there is a relatively high variability in results for epidemiological studies on thyroid diseases due to different factors such as: the population itself; the state of iodination of the population; the laboratory techniques used; or the reference values of thyroid hormones.^1^ To address this issue, the EUthyroid project (*Towards the elimination of iodine deficiency and related thyroid diseases in Europe*), of which our study is part, included, among other objectives, the comparison of the prevalence of different thyroid pathologies among the participating countries (data not yet published). This could give a global vision of how these pathologies are distributed in Europe and in turn relate them to the state of iodine nutrition in each country.

The prevalence of clinical hyperthyroidism (hyperT) varies between 0.2% and 1.3% in iodine‐sufficient areas of the world.^1^ In Europe and the United States, it is similar (0.7% vs. 0.5%, respectively), while in Australia it is slightly lower (0.3%). The highest rates of hyperT occur in iodine‐deficient countries, mainly due to the presence of toxic nodules in older patients. In the case of clinical hypothyroidism (hypoT) it is more prevalent and up to 10 times higher in women than in men. In Europe it ranges between 0.2%‐5.3% and in the United States between 0.3%‐3.7%, depending on the population studied.^1^

The use of pharmaceutical prescription records is a simple and relatively robust method for studying and monitoring the epidemiology of a specific treated pathology and also for comparing different populations. For this, the *defined daily dose* (DDD) can be used, which is the dose of a drug established for its main indication in non‐pregnant adult subjects. In the case of thyroid hormone treatment, the World Health Organization (WHO) specifies that DDD is 150 µg,^2^ a higher dose than the average dose currently consumed in Spain.^3^ To study of the prevalence of hypoT, the records of treated patients (NPT) maybe more useful. Similarly, diagnostic coding records from clinical practice allow an estimate of the prevalence of different pathologies to be made. There are no published data in Spain comparing the prevalence of hyperT and hypoT based on these two methods.

Thus, the objective of our study, framed within the EUthyroid Project, was to estimate the prevalence of hypoT and hyperT detected and treated during the period 2012‐2014 in Catalonia based on the records of pharmaceutical dispensing (PDR), and clinical diagnosis (DR) in 2014. A second objective was to compare the results obtained from the two sources.

## 2 MATERIAL AND METHODS

The population sample of the study was the whole population officially registered in the public health system of Catalonia (Catsalut) during the years 2012, 2013 and 2014. For the estimation of the prevalence, two databases were used. The one corresponding to the prescription and withdrawal of medication used in thyroid pathology (PDR) by the pharmaceutical office was obtained from the register of the Pharmaceutical Provision of the Catalan Health Service‐CatSalut; it contained the usual daily average dose of a drug (DDD). For this purpose, the HO3A codes, which define levothyroxine preparations, and the HO3B, which include antithyroid medication preparations were used. The number of patients under treatment (NPT) who had withdrawn the medication was also used. The NPT was used to calculate the prevalence, as it is a more suitable parameter than the DDD, which is used more in population calculations when individual data are not available.^3^ The analysis of the results was carried out by age and gender.

The second prevalence analysis was based on the diagnostic coding of thyroid pathology (DR) by recording the minimum basic set of primary care data (CMBD‐AP) of CatSalut in 2014. Data for 2012 and 2103 could not be included in the study because it was not available. The population base for that year was the same as in the first analysis. The following codes of the ninth edition of the International Classification of Diseases (ICD‐9) were chosen: 242 for hyperT and 243 and 244 for hypoT (Table 1). The analysis of the results was also performed by age and gender.

**Table 1:** ICD‐9 diagnostic codes used

Hypothyroidism	244.9 (Unspecified hypothyroidism, Hypothyroidism, primary or NOS, Myxedema, primary or NOS) 243 (Congenital hypothyroidism) 244.2 (Iodine hypothyroidism) 244.3 (Other iatrogenic hypothyroidism) 244.8 (Other specified acquired hypothyroidism Secondary hypothyroidism) 244.9 (Unspecified hypothyroidism, Hypothyroidism, primary or NOS)
Hyperthyroidism	242.0 (Toxic diffuse goitre) 242.00 (Toxic diffuse goitre without thyrotoxic crisis or storm) 242.01 (Toxic diffuse goitre with thyrotoxic crisis or storm) 242.1 (Toxic uninodular goitre) 242.2 (Toxic multinodular goitre) 242.20 (Toxic multinodular goitre without thyrotoxic crisis or storm) 242.21 (Toxic multinodular goitre with thyrotoxic crisis or storm) 242.3 (Toxic nodular goitre, unspecified) 242.30 (Toxic nodular goitre unspecified type without thyrotoxic crisis or storm) 242.4 (Thyrotoxicosis from ectopic thyroid nodule) 242.40 (Thyrotoxicosis from ectopic thyroid nodule without thyrotoxic crisis or storm) 242.80 (Thyrotoxicosis of other specified origin without thyrotoxic crisis or storm) 242.81 (Thyrotoxicosis of other specified origin with thyrotoxic crisis or storm) 242.9 (thyrotoxicosis without goitre or other cause) 242.90 (thyrotoxicosis without goitre or other cause and without thyrotoxic crisis or storm) 242.91 (Thyrotoxicosis without goitre or other cause with thyrotoxic crisis or storm)

Data from both registers were obtained with the authorization of the Pharmaceutical Provision of the Catalan Heath Service‐Catsalut and the Registry of the CMBD‐AP (Division of Demand Analysis and Activity‐Health Care Area) of the Government of Catalonia, respectively. Data of the Diagnostic Registers from the CMBD‐AP (Division of Demand Analysis and Activity‐Health Care Are) are fulfilled by Primary Care Physicians for the purpose of administrative and research tasks. In this register are included all citizens attended in public health care system, which in Spain is practically the whole population. People who are attended in private health care system are also attended in the public health system so they can have financed access to medicines. Registers don't include hospitals inpatients, but after discharge, they are followed‐up at Primary Care level, where the corresponding diagnostic is fulfilled by their general practitioner.

### 2.1 Statistical analysis

Categorical variables were expressed as a percentage. To compare the prevalence obtained by separating groups by gender, by the different years studied and between those obtained from the two different registries, the Chi‐square test or Fisher's exact test were used when appropriate. In all cases, *P* < .05 was considered to be statistically significant.

## 3 RESULTS

### 3.1 Data based on pharmaceutical dispensing

The prevalence of thyroid disorders was estimated from the pharmaceutical prescription database (Register of the Pharmaceutical Provision of the Catalan Health Service ‐ CatSalut). The population base in Catalonia in 2012 was 7 601 791 people, of which 213 271 subjects consumed 37 487 491.28 DDD of levothyroxine. The average dose of thyroid hormone consumption was 72 µg/day (0.488 DDD). In Table 2 population and consumption data for levothyroxine and antithyroid drugs for the years 2012, 2013 and 2014 according to the register of the Pharmaceutical Provision of the Catalan Health Service‐CatSalut are presented.

**Table 2:** Levothyroxine and antithyroid consumption in Catalonia during the years 2012‐2014 (pharmaceutical dispending register)

	2012	2013	2014	*P*
Insured population	7 601 791	7 568 982	7 556 330	‐
Levothyroxine (code H03A)				
NPT	213 271	221 373	231 975	<.005
HypoT Prevalence (based on NPT)	2.81%	2.92%	3.07%	<.005
DDD consumption	37 487 491.28	39 363 911.44	44 664 572.56	<.005
medium dose of LT4	72.23 µg/d	73.07 µg/d	77.35 µg/d	<.005
Antithyroid drugs (code HO3B)				
NPT	10 552	10 024	10 399	<.005
DDD consumption	1 277 056.67	1 217 493.34	1 275 830.00	<.005
Prevalence HiperT based on NPT	0.14%	0.13%	0.14%	NS

Abbreviations: DDD, defined daily dose; HyperT, hyperthyroidism; HypoT, hypothyroidism; NPT, number of patients under treatment; NS, non‐significant difference.

**Figure 1:**
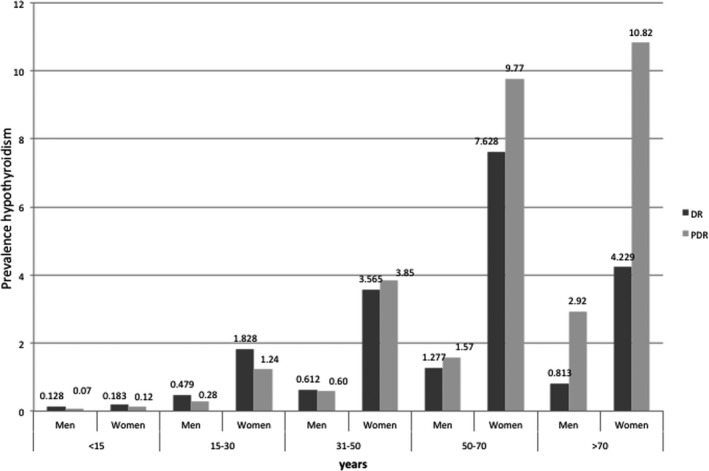
Comparison of the prevalence of hypothyroidism according to DR and PDR in 2014. In both registers, the prevalence is significantly different depending on age (*P* < .0001) and by gender in each age group (*P* < .0001). DR: Diagnosis registers, PDR: pharmacy dispensing registers

The overall prevalence of hypoT, measured by considering the NPT, increased significantly over this 3‐year period: 2.81% in 2012, 2.92% in 2013 and 3.07% in 2014 (*P* < .00001). The distribution was statistically different (*P* < .0001) by gender in the 3 years studied, with a much higher prevalence in women (0.81% vs 4.76% in 2012; 0.84% vs 4.96% in 2013 and 0.89% vs 5.19% in 2014). Likewise, a significantly higher prevalence of hypoT was observed as a function of age (*P* < .0001), presenting a similar pattern in the three years. (Figure 1).

**Figure 2:**
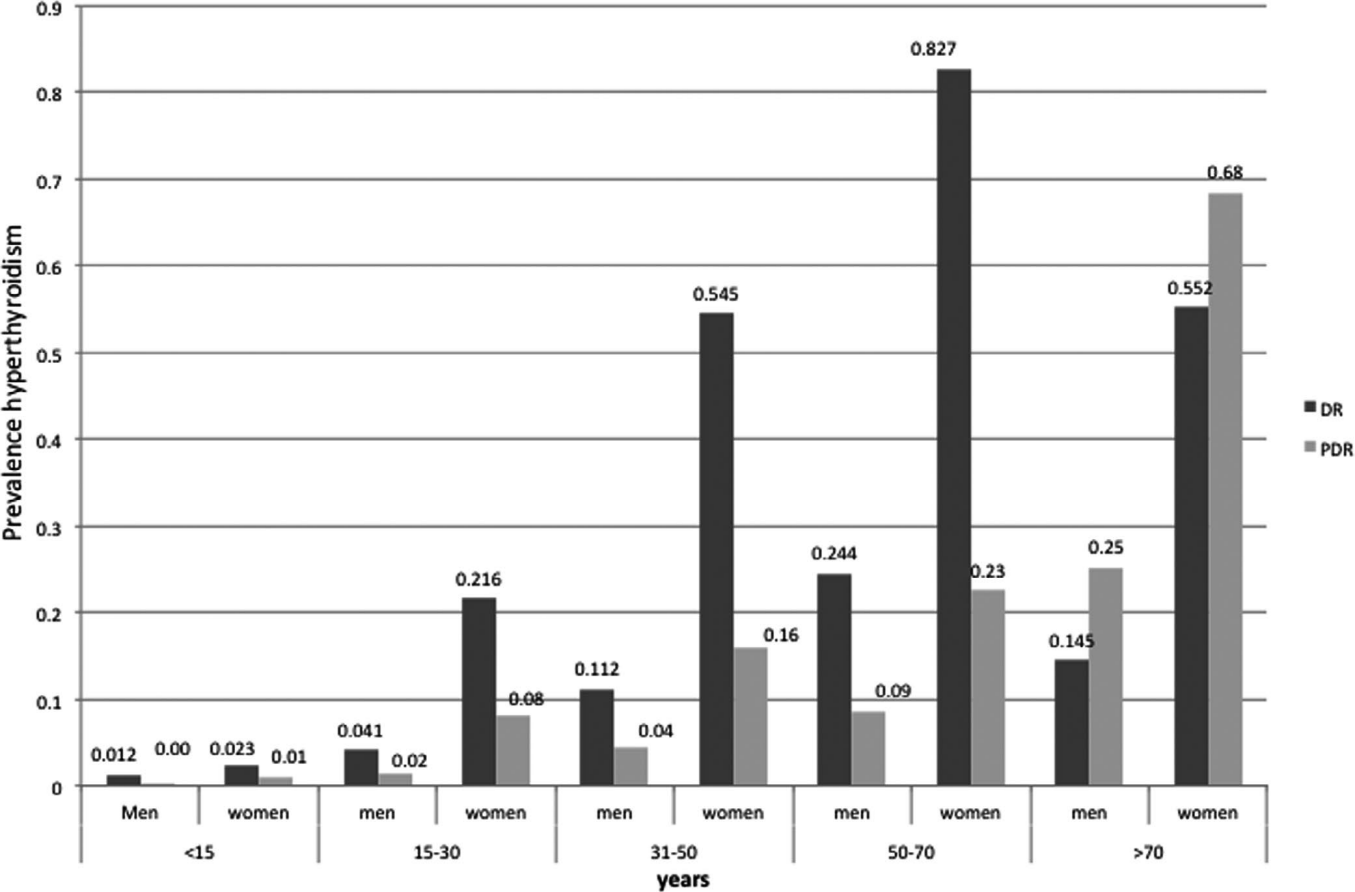
Comparison of the prevalence of hyperthyroidism according to DR and PDR in 2014. In both registers, the prevalence is significantly different depending on age (*P* < .0001) and by gender in each age group (*P* < .0001). DR: Diagnosis registers, PDR: pharmacy dispensing registers

The overall prevalence of hyperT according to the NPT showed no relevant changes over time: 0.14% in 2012, 0.13% in 2013 and 0.14% in 2014. The distribution of hyperT showed an increase with age and the prevalence was also higher in women (0.21% vs 0.06%; *P* < .0001) (Figure 2).

### 3.2 Data according to diagnostic record

The estimated prevalence of hypoT, according to the diagnostic records from considering codes 243 and 244 of the CMBD‐AP diagnostic register in 2014, was 2.54%. The distribution was statistically different (*P* < .0001) by gender, with a much higher prevalence in women (0.81% vs 4.23%) The prevalence of hyperT obtained by considering code 242 on the register was 0.35%. The prevalence was higher in women (0.145% vs 0.552%; *P* < .0001). The prevalence of hypoT and hyperT significantly increases with age (*P* < .0001), with a marked increase in people over 50 years old (Figure 1 and Figure 2).

### 3.3 Comparison of the two methods of estimation of thyroid disorders

When comparing the two methods used to estimate the prevalence of thyroid dysfunction in 2014, a higher prevalence of hypoT was found by using the pharmaceutical dispensing records, when compared to that estimated from the diagnostic registry (3.07% vs 2.54% *P* < .0001). However, when comparing the prevalence of hyperT obtained from the two methods, a markedly higher rate was found according to DR compared to that found by the pharmaceutical dispensing records (2.4% vs 0.14% *P* < .0001).

## 4 DISCUSSION

This is the first study carried out in Spain in which the prevalence of thyroid dysfunction was obtained and compared based on two different datasets: one from the PDR and the other from the DR. The differences observed are due in part to the fact that the estimated prevalence based on PDR includes those cases in which ‘clinical’ dysfunction was detected and therefore treatment established. When DR was the criteria, the prevalence estimation included cases of both ‘clinical’ and ‘subclinical’ dysfunction; and almost none, or very few of the subclinical cases received specific treatment. On the other hand hypoT is a chronic condition in most cases of life‐long duration, while hyperT is usually a transient condition, more or less long‐lasting and more or less recurrent in some cases, for which the treatment is obviously only administered during the active phases, but the diagnosis can persist actively on the register, even if the condition has been cured, or as in most cases, has ended up as hypoT.

In general, published studies regarding the epidemiology of thyroid dysfunction also show some heterogeneity in the results due to various factors including the methodology of the study performed, the population studied and the determination or not of thyroid antibodies or iodine status amongst others.^1^ The cross‐sectional studies on thyroid dysfunction performed in Spain, whether considering disorders of clinical or subclinical nature, show an overall prevalence between 8.9% and 12.3%.^4^, ^5^, ^6^ These figures are higher than those found in our study. A meta‐analysis ^7^ describes a prevalence of total thyroid dysfunction in Europe of 3.82%. This result is closer to that obtained in our study by using the PDR and quite different from what we found with the DR (3.2% vs 2.8%, respectively). Regarding total hypothyroidism (clinical and subclinical), the same study found a prevalence of 3.05% which is again similar to our result obtained according to PDR (3.07%) and higher than what we obtained by the DR (2.54%). There is probably an undercoding of both clinical and subclinical cases in our study. In the case of hyperthyroidism, this meta‐analysis shows a markedly higher prevalence (0.75%) than in our study, either when PDR is used (0.14%), which could be explained by the absence of subclinical pathology, which in many cases does not receive active treatment, or for DR data (0.35%) in which there could be again an undercoding bias phenomenon. Regarding the prevalence of undiagnosed thyroid dysfunction, the meta‐analysis of Garmendia et al^7^ found 6.71% (6.49‐6.93), where hypoT corresponded to a 4.94% and hyperT 1.72%. These data where not evaluable in our study.

Regarding the estimation of prevalence of thyroid dysfunction through the registration of diagnostic codes (DR), the Czech group Bilek et al,^8^ which also participates in the EUthyroid project, has described a prevalence of hypoT of 2.8% in 2012 and 3.2% in 2015 using code E03 (ICD‐10); in both years slightly higher than the prevalence observed in our study (2.54%). And in the case of the prevalence of hyperT, they found 0.7% in 2012 and 0.6% in 2015 through the code E05 (ICD‐10), results markedly higher than ours (0.35%).

Regarding drug consumption, our study shows a prevalence of hypoT of 2.81%‐3.07% in the time period studied, which is higher than that described in a similar study conducted in a Spanish population (Cádiz) by Escribano Serrano et al^3^ in 2014. The prevalence of hypoT in this later study using DDD was 1.24% (1.22‐1.27). They also used the DDP, defined as the *true average daily dose* that each patient takes when using a drug in its main indication adjusted by the DDD, and found a prevalence of hypoT of 2.39% (2.36‐2.43) and when the NPT was used the prevalence of hypoT was 2.86% (2.82‐2.90). The distribution by age and gender was very similar to that of our study, however they described a higher prevalence in women aged 50 to 70. The average daily dose of thyroxine was 75 mcg, similar to the 72 mcg obtained in our study. Morant et al^9^ studied the consumption of levothyroxine in Spain based on the DDP. The prevalence of hypoT was 0.32% and 0.43% in 1996 and 1999, respectively. The prevalence observed in Catalonia was 0.34% and 0.49%. Two other studies carried out in Spain observed a prevalence of hypoT of 1.4% in Valencia in 2003^10^ and 0.84% in Lleida, 2001,^11^ both cases based on the NPT. These results showed a markedly lower prevalence than the one in our study. These studies were carried out more than 10 years ago, therefore, the observed differences are likely due to underdiagnosis and lower iodine consumption. Another recent study conducted in a population of 66,843 inhabitants of the North East of England ^12^ showed an overall prevalence of hypoT based on the pharmaceutical prescription of 4.5%, with an age distribution similar to that of our study reaching a prevalence of 15.1% in patients over 90 years old. Our study observes an increase in use of levothyroxine over the years. This results are in line with observations from other Western countries, probably related to changes in indication for treatment as demonstrated by Medici et al.^13^ Our results show, for 2014, a different prevalence of both hyperthyroidism and hypothyroidism than the published data from Denmark,^14^ 0.14% vs 0.34% and 3.07% vs 1.92%, respectively. Probably, differences in iodine nutrition between the two countries explain these results. In the study by Rasmussen et al^15^ the median urinay iodine in the adult population of Denmark was 83 μg/L (years 2008‐2010), while in the adult population of Catalonia it was 147 μg/L in 2002.^16^

When comparing the prevalence of thyroid dysfunction obtained by the two methods used in our study, we observed that the prevalence of hypoT estimated by means of the PDR registries was higher than that estimated by the DR registry. The most feasible explanation for this is a diagnostic undercoding of hypoT, probably more pronounced in the age groups >50 years as the prevalence of this condition is known to be higher in this age group. In contrast, in the case of hyperT, a higher prevalence was found by DR than by PDR, probably due to the lack of inclusion of subclinical hyperT cases in these registries, due to the fact that in many cases they do not receive pharmacological treatment.

Our study has some limitations and some strengths. The description of the epidemiology of thyroid disorders remains a challenge and finding a robust methodology without bias that is validated and economically feasible remains a difficult task. The population evaluated in our study, which is part of the EUthyroid project, is the first one in which the two approaches, namely pharmaceutical dispensing records and diagnostic coding are compared in a Mediterranean population of more than 7 million inhabitants. However, both of these methods entail some biases. On the one hand, the PDR register does not usually include most of the cases of the subclinical forms of thyroid dysfunction, either hypoT or hyperT, since these subclinical situations often do not require pharmacological treatment. On the other hand, correct and exhaustive coding is limited by codes (ICD‐9 and IDD‐10) that are often confusing regarding the clinical classification of thyroid dysfunction. For example, it is not possible to distinguish between clinical and subclinical hyperthyroidism, and in the case of hypothyroidism only one subclinical category is included regarding iodine deficiency. Specific codes for hypothyroidism and subclinical hyperthyroidism would need to be included in the ‘International Classification of Diseases’. In addition to these difficulties, we can add the lack of time the doctors may suffer from, which can also contribute to undercoding.

In conclusion, our data indicate that the prevalence of thyroid dysfunction found are consistent with those published so far in iodine‐sufficient populations, where the prevalence of hypoT is relatively high, reaching almost 10% in women over 50 and the prevalence of hyperT is low, although it increases with age and in women. Likewise, our study supports the use of the pharmacy dispensing and diagnosis registries for the estimation of the whole population prevalence of thyroid disorders. This is because, despite the inherent biases they present, they could be performed continuously over time and are more feasible than other methods such as cross‐sectional studies, which although more precise, require a strong and costly organizational effort.

## CONFLICT OF INTEREST

None of the authors have conflict of interest related to this article.

## AUTHOR CONTRIBUTIONS

LV and MP initiated and coordinated the study. ST and LV conceptualized and planned the analyses. MM performed the statistical analysis. ST, LV, BS and MP contributed to the preparation of the manuscript and data interpretation. All authors reviewed, revised and approved the final manuscript.

## ETHICS APPROVAL

This study is included in the EUthyroid Project which complies with the necessary ethical principles to be accepted by the Horizon 2020 research and innovation programme of the European Union, as set out in the European Code of Conduct for Research Integrity. The analysed databases follow the legal criteria to guarantee the confidentiality of personal information. Legal and ethical conditions to consult these database are developed in this address: http://web.gencat.cat/ca/menu-ajuda/ajuda/avis_legal/.

## Data Availability

The data that support the findings of this study are available from “Register of the Pharmaceutical Provision and “Register of the CMBD‐AP of Catalan Health Service. Restrictions apply to the availability of these data, which were used under license for this study. Data are available from http://medicaments.gencat.cat/ca/contacte/ and https://catsalut.gencat.cat/ca/proveidors-professionals/registres-catalegs/registres/cmbd/ with the permission of Catalan Health Service.
